# Mangrove Extraction Algorithm Based on Orthogonal Matching Filter-Weighted Least Squares

**DOI:** 10.3390/s24227224

**Published:** 2024-11-12

**Authors:** Yongze Li, Jin Ma, Dongyang Fu, Jiajun Yuan, Dazhao Liu

**Affiliations:** School of Electronics and Information Engineering, Guangdong Ocean University, Zhanjiang 524088, China; 14767622730@stu.gdou.edu.cn (Y.L.); 2112210027@stu.gdou.edu.cn (J.M.); fudy@gdou.edu.cn (D.F.); 18973232665@stu.gdou.edu.cn (J.Y.)

**Keywords:** mangroves, target extraction, GF-6 remote sensing images, edge detection

## Abstract

High-precision extraction of mangrove areas is a crucial prerequisite for estimating mangrove area as well as for regional planning and ecological protection. However, mangroves typically grow in coastal and near-shore areas with complex water colors, where traditional mangrove extraction algorithms face challenges such as unclear region segmentation and insufficient accuracy. To address this issue, in this paper we propose a new algorithm for mangrove identification and extraction based on Orthogonal Matching Filter–Weighted Least Squares (OMF-WLS) target spectral information. This method first selects GF-6 remote sensing images with less cloud cover, then enhances mangrove feature information through preprocessing and band extension, combining whitened orthogonal subspace projection with the whitened matching filter algorithm. Notably, this paper innovatively introduces Weighted Least Squares (WLS) filtering technology. WLS filtering precisely processes high-frequency noise and edge details in images using an adaptive weighting matrix, significantly improving the edge clarity and overall quality of mangrove images. This innovative approach overcomes the bottleneck of traditional methods in effectively extracting edge information against complex water color backgrounds. Finally, Otsu’s method is used for adaptive threshold segmentation of GF-6 remote sensing images to achieve target extraction of mangrove areas. Our experimental results show that OMF-WLS improves extraction accuracy compared to traditional methods, with overall precision increasing from 0.95702 to 0.99366 and the Kappa coefficient rising from 0.88436 to 0.98233. In addition, our proposed method provides significant improvements in other metrics, demonstrating better overall performance. These findings can provide more reliable technical support for the monitoring and protection of mangrove resources.

## 1. Introduction

Mangrove forests are a valuable resource that have received extensive international research attention. Current studies cover a wide range of topics, including biodiversity, ecosystem services, and the impact of global change on mangroves. The protection and management of mangroves have become a common concern of the international community. Governments and organizations worldwide are taking measures to protect, restore, and effectively utilize mangrove ecosystems, contributing to the international commitment to address climate change and promote sustainable resource use [1].

Mangrove ecosystems, as one of the most important coastal-interaction ecosystems, are crucial for maintaining marine biodiversity and ecological balance in the ocean [2]. Mangroves often grow in coastal areas that are close to frequent human activities, and the development of such activities can bring about a series of negative impacts on the surrounding mangrove ecosystems. This has attracted attention from both the academic community and regulatory bodies. For example, activities in coastal aquaculture zones may lead to water pollution in the surrounding area, disrupting the stability of mangrove ecosystems. Land reclamation and other similar activities may also disrupt the balance of marine ecosystems, affecting the survival and reproduction of flora and fauna around mangroves. These human activities can impact the biodiversity and ecological functions of mangroves, leading to changes in land use, habitat loss, and degradation of mangrove areas.P attanaik C. et al. [3] found that in 2006 the Mahanadi delta in the eastern Indian state of Odisha featured dense mangrove forest (12.6%), open mangrove forest (3.3%), aquaculture (12.9%), and agriculture (30.9%); however, between 1973 and 2006 the mangrove area decreased by 2606 hectares, while the aquaculture area increased by 3657 hectares, clearly illustrating the growth of the aquaculture industry.

In order to achieve sustainable development of mangrove areas, it is necessary to strengthen management and supervision, adopt scientific environmental protection measures, ensure the positive interaction of mangroves, protect the marine ecological environment, and achieve sustainable resource utilization [4]. In addition, timely monitoring and regulation of mangrove ecosystems is crucial for their effective protection. However, traditional manual inspections and monitoring methods are currently too slow and require multiple field measurements, consuming significant human and material resources. Today, this approach is gradually failing to meet growing monitoring demands. In recent years, the development of remote sensing technology has increased the accuracy of optical sensors, leading to remote sensing gradually becoming one of the main monitoring methods thanks to its advantages of large-scale coverage, short periodicity, real-time monitoring, and low cost. For ecosystems in natural reserves, including mangroves, monitoring via remote sensing offers unique advantages. By leveraging the continuity of sensor systems and image acquisition capabilities, remote sensing can help to quickly obtain spatial distribution information on mangrove regions [5]. Remote sensing image object detection is an important branch of remote sensing data processing that has seen the development of many detection algorithms, including Spectral Angle Mapper (SAM), Orthogonal Subspace Projection (OSP), Matched Filter (MF), and Constrained Energy Minimization (CEM), among others [6,7,8,9].

In recent years, many scholars have conducted extensive research and exploration on the extraction and detection of mangroves based on remote sensing data. Pacheco et al. [10] evaluated the greenhouse gas flux emissions from the soil of dead and healthy mangroves in southeastern Brazil, while Jia M et al. [11] conducted an analysis of the global mangrove status from different perspectives and generated the first 10 m resolution dataset of global mangroves, providing a key reference for evaluating the role of mangroves in sustainability and assessing sustainable development goals. In [12], Zhang J et al. analyzed the spatiotemporal changes in mangrove landscape patterns in China over the past 40 years based on remote sensing data.

Although manual visual interpretation can yield accurate results, it is costly, inefficient, and unable to effectively handle large-scale data. In addition, manual inspection encounters limitations such as inability to process data in real-time and susceptibility to human error. On the other hand, while neural network-based methods have made significant progress in recent years, these methods typically require large-scale datasets for thorough training in practical applications. Existing training datasets are often limited to specific regions or specific types of crops, and may be insufficiently representative or lacking in coverage. The limitations of existing datasets severely affect the generalization capability and accuracy of the resulting models. Furthermore, the quality and accuracy of dataset annotations are also critical factors affecting the performance of neural network models. Remote sensing data present specific challenges in mangrove monitoring, such as variable water color, cloud cover, and the need for high spatial resolution. Variations in water color affect the spectral characteristics of mangroves, making it difficult for single spectral analysis methods to accurately identify mangroves, while cloud cover can obscure ground information, affecting data completeness and availability. The demand for high spatial resolution further increases the complexity and computational cost of data processing.

Therefore, this paper presents a new algorithm called Orthogonal Matching Filter–Weighted Least Squares (OMF-WLS), which combines the Orthogonal Subspace Projection (OSP), Matched Filter (MF), and Weighted Least Squares (WLS) methods for mangrove identification based on GF-6 satellite remote sensing imagery. The goal is to develop a mangrove extraction method that is efficient, simple, reliable, and accurate for images with complex backgrounds. By addressing the limitations of existing technologies and the specific challenges posed by remote sensing data, this new algorithm can provide a powerful tool for mangrove monitoring.

## 2. Research Region and Data

This section introduces the mangrove areas selected for the study and the sources of satellite imagery data.

### 2.1. Overview of the Study Area

**Figure 1:**
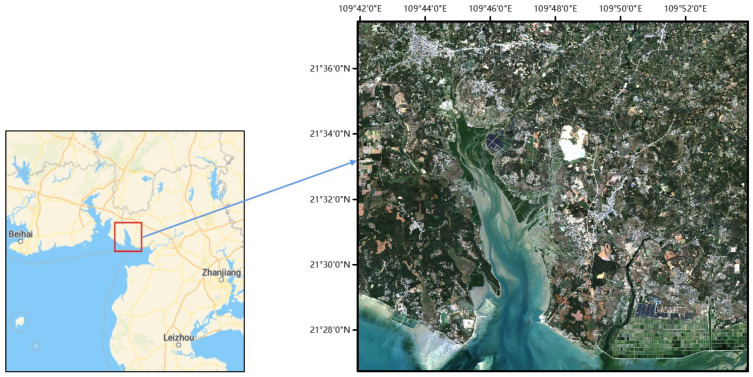
Study area (Yingluo Port).

The area selected for this study is located in the Yingluo Port area of Shankou Town, Hepu County, Guangxi Zhuang Autonomous Area, China. This area was designated as a national nature reserve in 1990, with the primary aim of protecting mangroves. The area has a South Asian subtropical maritime climate characterized by abundant sunlight and heat resources and ample rainfall, with an average annual temperature of 22.9 °C. The extreme maximum temperature is 38.2 °C and the extreme minimum temperature is 1.5 °C. The area is frost-free throughout the year, with 80–85% of the annual precipitation concentrated from April to September, and the cumulative average annual precipitation is 1573.4 mm. The study focuses specifically on the mangroves in this area, which feature many well-developed, structurally typical, large, contiguous, and relatively intact natural mangroves. There are at least twelve species of mangrove plants holding significant scientific value. Identifying and extracting mangrove areas is crucial for maintaining the health and sustainable development of the ecosystem, and monitoring this area is the primary focus of the present study. Figure 1 shows the Yingluo Port area.

### 2.2. Data Sources

The remote sensing images used in this study are from the GF-6 satellite data. The GaoFen-6 (GF-6) satellite was developed by the China Aerospace Corporation (CASC) and successfully launched on 2 June, 2018. It is a low-orbit optical imaging remote sensing satellite equipped with a 2-m panchromatic/8-m multispectral high-resolution camera and a 16-m multispectral medium-resolution wide-field camera. The 2-m panchromatic/8-m multispectral camera has an observation width of 90 km, while the 16-m multispectral camera has an observation width of 800 km [13]. This paper uses GF-6 data purchased from the official website, which is open for use and solely intended for scientific research. The satellite data from the GF-6 satellite’s 2-m panchromatic/8-m multispectral high-resolution camera were acquired on 30 January 2023. These data include five bands: panchromatic, blue, green, red, and near-infrared. The cropped Yingluo Port area was preprocessed using ENVI 5.6 software. To further enhance the resolution, image fusion was performed by combining the panchromatic band with the multispectral bands using the Gram–Schmidt (GS) method. This process resulted in a multispectral image with a resolution of 2 m [14]. As this paper focuses on mangrove identification, the multispectral images were subjected to land–sea separation to exclude interference from the ocean. This process involved editing vector features using ArcGIS and was followed by mask processing.

## 3. Processing Flow and Algorithm Framework

This section outlines the basic process for mangrove identification, describes the band expansion before algorithm processing, and assesses the effectiveness of Otsu’s method for threshold segmentation after algorithm processing. Additionally, it explains the primary principles of the OSP and MF algorithms, then introduces our new algorithm combining these principles. This new algorithm incorporates optimization techniques such as whitening and regularization. Finally, the solution process of the OMF-WLS algorithm is thoroughly derived under rigorous mathematical theory.

### 3.1. Technical Route

**Figure 2:**
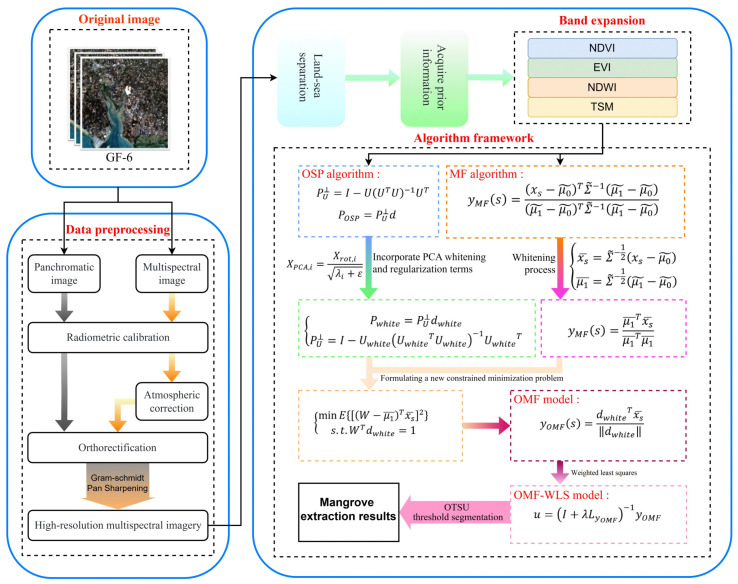
Extraction process of mangrove remote sensing imagery based on OMF-WLS.

To achieve mangrove identification and extraction, this paper proposes the new OMF-WLS algorithm. The innovation of this algorithm lies in its integration of OSP, MF, and WLS. By introducing whitening processing, regularization, and weighted least squares filtering, the algorithm enhances target information and weakens background information. Finally, Otsu’s method is used for threshold segmentation, effectively enabling precise extraction of the target. The specific extraction process is shown in Figure 2.

### 3.2. Band Expansion

The Remote Sensing Ecological Index (RSEI) is a technique used in remote sensing image processing and analysis [15]. It involves combining different spectral bands from satellite multispectral imagery to construct and enhance spectral characteristics, thereby reflecting the features of a specific land cover [16]. In order to achieve better extraction of mangroves, this paper utilizes a method based on constructing feature indices to build and enhance the target spectral characteristics to reflect and extract the land cover features of mangroves. These feature indices are typically derived from new image data obtained by mathematical operations and combinations of different spectral bands in remote sensing images, which are used to reflect certain attributes or conditions of the Earth’s surface. The extended feature indices constructed in this paper are mainly divided into two categories. First, the vegetation index category primarily reflects the growth status and coverage of vegetation through the combination of infrared and visible light bands. Common vegetation indices include the Normalized Difference Vegetation Index (NDVI), among others [17]. These indices can effectively distinguish mangroves from background information, as mangroves typically have high vegetation cover. Second, the water index category primarily reflects the distribution and types of water bodies through combination of the blue light and near-infrared bands. Common water indices include the Normalized Difference Water Index (NDWI), among others [18]. These indices allow for effective differentiation between water bodies and land areas, as water indices typically have higher values in water bodies and lower values in land areas.

Because the PCA whitening in our algorithm is implemented based on Singular Value Decomposition (SVD), we select only four indices in order to avoid the presence of highly correlated features in the input data, as more indices could lead to singular values close to zero in the SVD results and could even result in overfitting [19,20,21]. The four indices selected to differentiate mangrove areas are the Normalized Difference Vegetation Index (NDVI), Enhanced Vegetation Index (EVI), Normalized Difference Water Index (NDWI), and Total Suspended Matter (TSM) [22,23,24]. After band extension, tis results in eight bands. As shown in Table 1, these bands represent the characteristic indices after band extension, where *B*1 represents the blue band, *B*2 represents the green band, *B*3 represents the red band, and *B*4 represents the near-infrared band.

### 3.3. Traditional Algorithms

#### 3.3.1. Orthogonal Subspace Projection Method

Orthogonal Subspace Projection (OSP) is a commonly used technique for feature extraction and dimensionality reduction, particularly in fields such as signal processing, pattern recognition, and data analysis [25,26].

First, let *X* be a L×1 pixel spectral vector and let *K* represents the dimension of the end-member spectral matrix, where $\alpha_{i}$ represents the abundance corresponding to the end-member. The expression is

(1) $$\left\{\begin{array}{c} \alpha_{i}\geq 0,\;1\leq i\leq K\\ \sum_{i=1}^{k}\alpha_{i}=1. \end{array}\right.$$

The OSP model is composed of the target signal *d* and the background end-members *U*, which can be expressed as

(2) $$X=d_{\alpha_{d}}+U_{\alpha_{U}}+n.$$

In Equation (2), $d_{\alpha_{d}}$ represents the abundance vector of the target signal, $U_{\alpha_{U}}$ represents the abundance vector of the background end-members, and *n* denotes the noise or model error. It should be noted that $\alpha_{d}$ is a scalar, while $\alpha_{U}$ is a vector. The dimension of *U* is L×(K−1) and the dimension of $\alpha_{U}$ is (K−1)×1. Thus, the result of $U_{\alpha_{U}}$ is a vector of dimension L×1.

In order to extract the target signal of interest while suppressing all other background features, an orthogonal subspace projector is introduced, with ⊥ denoting the orthogonal complement space, defined as follows:

(3) $$P_{U}^{⊥}=I-U{\left(U^{T}U\right)}^{-1}U^{T}.$$

The constructed filter detector can be expressed as

(4) $$P_{OSP}=P_{U}^{⊥}d.$$

In Equation (3), *I* is the L×L identity matrix, $U^{T}U$ is a (K−1)×(K−1) matrix, and ${\left(U^{T}U\right)}^{-1}U^{T}$ is a (K−1)×L matrix. Therefore, $U{\left(U^{T}U\right)}^{-1}U^{T}$ is also an L×L matrix, meaning that the entire projector $P_{U}^{⊥}$ has dimensions L×L, while ${\left(U^{T}U\right)}^{-1}U^{T}$ is the pseudoinverse of the matrix *U*, with $U^{T}$ representing the transpose of matrix *U*. As shown in Equation (4), prior knowledge of the image end-members is required for end-member extraction.

#### 3.3.2. Matched Filter

A Matched Filter (MF) is a signal processing filter that maximizes the signal-to-noise ratio at a specific time. The MF allows for the effective extraction of the target signal [27,28]. Matched filters in image object detection help to extract specific features of the target object and find areas in the image that match the template signal, thereby enabling object detection functionality.

Assuming that the image to be analyzed has *B* bands, the vector form of each pixel can be expressed as

(5) $$x_{s}={\left[x_{1},\;x_{2},\;x_{3},\;\cdots ,\;x_{B}\right]}^{T}\left(s=1,\;2,\;3,\;\cdots ,\;S\right).$$

In Equation (5), S=LENGTH×WIDTH, where *LENGTH* is the length of the image dimension and *WIDTH* is the width of the image dimension. Each band’s sensor obtains a grayscale image $G_{b}$ with size *S*:

(6) $$G_{b}\left(length,\;width\right)\left(b=1,\;2,\;3,\;\cdots ,\;B;\;length=1,\;2,\;3,\;\cdots ,\;LENGTH;\;width=1,\;2,\;3,\;\cdots ,\;WIDTH\right).$$

Three-dimensional imaging data can be rearranged as

(7) $$X=[x_{1},\;x_{2},\;x_{3},\;\cdots ,\;x_{S}].$$

In Equation (2), *X* focuses on how the spectral vectors (such as those from each pixel) are decomposed into the three components of target, background, and noise. In Equation (7), *X* is more macroscopic, representing the spectral characteristics of the entire dataset. These are different representations of the same data; as such, this can be expressed as

(8) X(b, s)(b=1, 2, 3, ⋯, B; s=1, 2, 3, ⋯, S).

The relationship between Equations (6) and (8) is as follows:

(9) $$G_{b}\left(length,width\right)=X\left(b,\;\left(length-1\right)WIDTH+width\right).$$

Based on the known prior information, this can be classified as a simple quadratic programming problem:

(10) $$\left\{\begin{array}{c} \min W^{T}\sum W\\ s.t.W^{T}\left(\mu_{1}-\mu_{0}\right)=1. \end{array}\right.$$

The mean vector for the target pixel is denoted as $\mu_{1}$ and the mean vector for the background pixel as $\mu_{0}$, while *W* is the linear filter. By introducing the Lagrange multiplier method, we can solve for the unknown vector *W*:

(11) $$W=\frac{\Sigma^{-1}\left(\mu_{1}-\mu_{0}\right)}{{\left(\mu_{1}-\mu_{0}\right)}^{T}\Sigma^{-1}\left(\mu_{1}-\mu_{0}\right)}.$$

The covariance matrix $\tilde{\Sigma }$ and the background mean vector $\tilde{\mu_{0}}$ can be estimated based on the existing data. Combined with Equation (11), the form of the linear detector obtained is

(12) $$y_{W}=W^{T}\left(x_{i}-\tilde{\mu_{0}}\right).$$

The target mean vector is obtained from the average of the existing spectral library or label information:

(13) $$\tilde{\mu_{1}}\approx \frac{1}{S}\sum_{j=0}^{s}x_{j}.$$

Combining the data from Equations (11)–(13) and performing the Mahalanobis distance calculation between the target and the background, we finally obtain

(14) $$y_{MF}\left(s\right)=\frac{{\left(x_{s}-\tilde{\mu_{0}}\right)}^{T}{\tilde{\Sigma }}^{-1}\left(\tilde{\mu_{1}}-\tilde{\mu_{0}}\right)}{{\left(\tilde{\mu_{1}}-\tilde{\mu_{0}}\right)}^{T}{\tilde{\Sigma }}^{-1}\left(\tilde{\mu_{1}}-\tilde{\mu_{0}}\right)}.$$

### 3.4. Orthogonal Matching Filter-Weighted Least Squares Algorithm

#### 3.4.1. Data Preprocessing

Before using algorithms for target information enhancement and whitening, the image data are first subjected to two-dimensionalization and normalization. This involves converting three-dimensional multispectral images into two-dimensional matrices and mapping the data to a range between 0 and 1. This step removes the dimensionality and units of measurement of the different dimensions of the data. Subsequently, the data undergo mean normalization to center them around 0, followed by standardization to ensure that the features are all on the same scale.

As the data have been mean-normalized, the means $\bar{x}=\bar{y}=0$. When we focus on the dataset of *X*, the covariance matrix can be simplified to Equation (15):

(15) $$C=\frac{1}{n}\sum_{i=1}^{n}x_{i}{x_{i}}^{T}.$$

Then, we perform eigenvalue decomposition on the covariance matrix *C* to compute the eigenvalues and eigenvectors, which are

(16) $$C=U\Lambda U^{T}.$$

In Equation (16), *U* represents the eigenvectors and Λ is the diagonal matrix with the eigenvalues on the diagonal.

Whitening is a linear transformation used to decorrelate source signals, and is an important preprocessing step [29]. Its purpose is to reduce the redundancy in the input data to ensure that the whitened input data have the following properties:

1. No correlation between features.
2. The variance of all features is equal to 1.
3. Reduced dimensionality.

The implementation of PCA whitening is similar to PCA dimensionality reduction [30]. After obtaining the eigenvalues and eigenvectors of the covariance, the transpose of the eigenvector matrix is left-multiplied by the original data matrix to achieve a rotational transformation of the data. Then, each dimension of the transformed data matrix is divided by the corresponding variance (i.e., eigenvalue).

#### 3.4.2. Algorithm Flow

By scaling our decorrelated data through dividing each feature $x_{rot,i}$ by the square root of its corresponding eigenvalue $\sqrt{\lambda_{i}}$, we obtain the PCA whitened [31] data matrix:

(17) $$X_{PCA,i}=\frac{X_{rot,i}}{\sqrt{\lambda_{i}}}.$$

Sometimes, the eigenvalues $\lambda_{i}$ may be very close to zero. This can cause numerical instability or overflows when dividing by values close to zero. To address this issue, the eigenvalues are scaled and a very small constant ε is added to avoid division by values close to zero (typically $\varepsilon \approx 10^{-5}$). This constant is commonly referred to as a regularization term. By adding this constant, numerical instability and overflows during division operations can be avoided. Thus, the final result of the PCA whitening matrix is as follows:

(18) $$X_{PCA,i}=\frac{X_{rot,i}}{\sqrt{\lambda_{i}+\varepsilon }}.$$

We combine Equations (2)–(4) with Equation (18) to perform whitening weighting operations:

(19) $$\left\{\begin{array}{c} d_{white}={\left(d^{T}X_{PCA,i}\right)}^{T}\\ U_{white}={\left(U^{T}X_{PCA,i}\right)}^{T}. \end{array}\right.$$

Therefore, the detector can be redefined as

(20) $$\left\{\begin{array}{c} P_{white}=P_{U}^{⊥}d_{white}\\ P_{U}^{⊥}=I-U_{white}{\left(U_{white}^{T}U_{white}\right)}^{-1}U_{white}^{T}. \end{array}\right.$$

Finally, a sparse matrix is constructed from the orthogonal subspace projector $P_{U}^{⊥}$ in Equation (20), which effectively enhances the target information and suppresses the background information.

Based on the whitened sample space, we have

(21) $$\left\{\begin{array}{c} \bar{x_{s}}={\tilde{\Sigma }}^{-\frac{1}{2}}\left(x_{s}-\tilde{\mu_{0}}\right)\\ \bar{\mu_{1}}={\tilde{\Sigma }}^{-\frac{1}{2}}\left(\tilde{\mu_{1}}-\tilde{\mu_{0}}\right). \end{array}\right.$$

Therefore, the MF can be rewritten as

(22) $$y_{MF}\left(s\right)=\frac{{\bar{\mu_{1}}}^{T}\bar{x_{s}}}{{\bar{\mu_{1}}}^{T}\bar{\mu_{1}}}.$$

where ${\bar{\mu_{1}}}^{T}\bar{\mu_{1}}$ is a constant. A new measurement method can be used to find the projection vector *W*. In the whitened space, *W* can be found by minimization, resulting in a new output

(23) $$y_{MF}\left(s\right)=W^{T}\bar{x_{s}}.$$

Using $d_{white}$ in Equation (19) as the target vector $\bar{\mu_{1}}$ in the matching filter, a new Orthogonal Matching Filter (OMF) optimization model is formed. The constrained minimization problem then becomes

(24) $$\left\{\begin{array}{c} \min E\{{\left[{\left(W-\bar{\mu_{1}}\right)}^{T}\bar{x_{s}}\right]}^{2}\}\\ s.t.W^{T}d_{white}=1. \end{array}\right.$$

Expanding and simplifying this model results in

(25) $$\left\{\begin{array}{c} \min W^{T}\Sigma_{\mathrm{OMF}}W-2W^{T}\Sigma_{\mathrm{OMF}}\bar{\mu_{1}}+{\bar{\mu_{1}}}^{T}\Sigma_{\mathrm{OMF}}\bar{\mu_{1}}\\ s.t.\;W^{T}d_{\mathrm{white}}=1. \end{array}\right.$$

Here, $\Sigma_{\mathrm{OMF}}$ represents the autocovariance matrix of the whitened samples after weighting. In the whitened space, the covariance matrix is the identity matrix. Solving Equation (25) yields

(26) $$W=\frac{d_{white}}{∥d_{white}∥}.$$

Therefore, the final output of the OMF is

(27) $$y_{OMF}\left(s\right)=\frac{{d_{white}}^{T}\bar{x_{s}}}{∥d_{white}∥}.$$

#### 3.4.3. Weighted Least Squares Filtering Algorithm

The Weighted Least Squares (WLS) filtering algorithm is commonly used in image processing for edge preservation and noise reduction. The main purpose of applying the WLS algorithm before threshold segmentation is to smooth the image while preserving as much edge information as possible, thereby improving the quality and accuracy of the segmentation [32]. Specifically, the WLS algorithm performs weighted smoothing on the image by combining the gradient information with the spatial relationships between pixels. This approach reduces noise in the image while preserving edge information.

Applying the WLS algorithm before threshold segmentation helps to improve image quality, allowing subsequent segmentation algorithms to more accurately distinguish between objects and background in the image. This aids in extracting clear and precise edges, making it easier for the threshold segmentation algorithm to perform segmentation near edges and reducing segmentation errors caused by noise or blurring.

We perform WLS filtering on the image after it has undergone whitening and weighted filtering:

(28) $$\min_{u}{\left(u-y_{OMF}\right)}^{T}\left(u-y_{OMF}\right)+\lambda \left(u^{T}D_{x}^{T}A_{x}D_{x}u+u^{T}D_{y}^{T}A_{y}D_{y}u\right).$$

In Equation (28), *u* represents the filtered image to be obtained and λ is the regularization parameter used to balance the tradeoff between *u* and $y_{OMF}$. When the gradient changes at the edges of the input image are significant, we want to impose a smaller constraint to preserve the structural information of the image; conversely, when the gradient changes at the image edges are minimal, these details are considered less important and a larger constraint can be applied. Here, $D_{x}$ and $D_{y}$ are discrete difference operators, while $A_{x}$ and $A_{y}$ are diagonal matrices. By differentiating the above Equation (28), the following minimum value solution for *u* is obtained:

(29) $$u={\left(\begin{array}{c} I+\lambda L_{y_{OMF}} \end{array}\right)}^{-1}y_{OMF}$$

where $L_{y_{OMF}}=D_{x}^{T}A_{x}D_{x}+D_{y}^{T}A_{y}D_{y}$ is a Laplacian matrix. Thus, Equation (29) represents the final output of the Orthogonal Matching Filter–Weighted Least Squares (OMF-WLS) algorithm presented in this paper.

### 3.5. Otsu’s Algorithm

Otsu’s method is a classic algorithm used in image processing and computer vision for image thresholding. Otsu’s algorithm aims to automatically determine the threshold for binarizing an image such that the segmented image has the optimal between-class variance [33]. In this paper, the image filtered using Equation (31) is binarized and segmented based on Otsu’s method.

Otsu’s algorithm divides image pixels into two categories, namely, the object and the background. Supposing that the image has *L* grayscale levels, where 0<T<L−1, let $p_{1}$ be the proportion of object pixels to the total number of pixels in the image and let $p_{2}$ be the proportion of background pixels to the total number of pixels. Additionally, let $m_{1}$ be the mean grayscale value of the object pixels and let $m_{2}$ be the mean grayscale value of the background pixels. Then, the global grayscale mean *G* of the image is provided by

(30) $$G=m_{1}p_{1}+m_{2}p_{2},$$

while the between-class variance $\sigma^{2}$ is provided by

(31) $$\sigma^{2}=p_{1}{\left(m_{1}-G\right)}^{2}+p_{2}{\left(m_{2}-G\right)}^{2}.$$

Combined with Equation (31), this simplifies to

(32) $$\sigma^{2}=p_{1}p_{2}{\left(m_{1}-m_{2}\right)}^{2}.$$

Finally, by iterating through all grayscale levels, the optimal threshold $T^{*}$ that maximizes the value of $\sigma^{2}$ is determined as follows:

(33) $$\sigma^{2}\left(T^{*}\right)=\max_{0<T<L-1}\sigma^{2}.$$

This algorithm can quickly and automatically select the optimal threshold for segmentation, allowing for accurate extraction of targets such as mangroves.

## 4. Results Analysis and Accuracy Evaluation

This section introduces the various metrics used to evaluate the extraction accuracy of mangroves. The OMF-WLS algorithm presented in this paper is compared with other methods by selecting different mangrove study areas. The purpose of the comparison is to assess the advantages of the proposed algorithm in mangrove extraction.

For the large-scale images in this paper with a spatial resolution of 2 m, the time required to run the OMF-WLS algorithm in Matlab R2020a was 25.0197 s in Area A, 8.2723 s in Area B, and 1.9815 s in Area C. These times do not consider the time spent to preprocess the images using ENVI software. It is worth noting that all experiments were conducted on a computer with Windows 10, AMD Ryzen 5 5600 G with Radeon Graphics 3.90 GHz, and 16 GB of RAM.

### 4.1. Detection Accuracy Metrics

The training set used in this study was derived from randomly sampled multiple sample points in the mangrove area shown in Figure 1. The validation set consisted of randomly selected sample points from Figure 1, which included both mangrove and non-mangrove areas. The test set involved selecting three mangrove areas from Figure 1 with different sizes for recognition and evaluation.

To better quantify and assess the algorithm’s ability to detect and extract mangrove areas, we randomly selected ten samples from the research area of Yingluo Port. These samples represented a wide range of mangrove ecosystem characteristics, and the selection of ten samples helped to avoid erroneous choices. The average of these samples was used to obtain prior information about the target, after which a spectral library was exported using ENVI. Based on the selected sample data, a confusion matrix was then used to calculate four metrics for mangrove detection: Overall Accuracy (OA), Average Accuracy (AA), Producer’s Accuracy (PA), and Kappa coefficient [34,35]. The formulas for these metrics are shown below.

(34) $$\left\{\begin{array}{l} \mathrm{OA}=\frac{\mathrm{TP}+\mathrm{TN}}{\mathrm{TP}+\mathrm{TN}+\mathrm{FP}+\mathrm{FN}}\\ \mathrm{AA}=\frac{1}{2}\left(\frac{\mathrm{TP}}{\mathrm{TP}+\mathrm{FN}}+\frac{\mathrm{TN}}{\mathrm{TN}+\mathrm{FP}}\right)\\ \mathrm{PA}=\frac{\mathrm{TP}}{\mathrm{TP}+\mathrm{FP}}\\ \mathrm{Kappa}=\frac{p_{0}-p_{e}}{1-p_{e}}\left\{\begin{array}{c} p_{0}=\frac{\mathrm{TP}+\mathrm{TN}}{\mathrm{TP}+\mathrm{TN}+\mathrm{FP}+\mathrm{FN}}\\ p_{e}=\frac{\left(\mathrm{TP}+\mathrm{FP})(\mathrm{TP}+\mathrm{FN})+(\mathrm{FN}+\mathrm{TN})(\mathrm{FP}+\mathrm{TN}\right)}{{\left(\mathrm{TP}+\mathrm{TN}+\mathrm{FP}+\mathrm{FN}\right)}^{2}} \end{array}\right. \end{array}\right.$$

In Formula (34), TP (True Positive) represents the number of true positive examples, when the model predicts the positive class and the actual label is also positive; TN (True Negative) represents the number of true negative examples, when the model predicts the negative class and the actual label is also negative; FP (False Positive) represents the number of false positive examples, when the model predicts the positive class but the actual label is negative; and FN (False Negative) represents the number of false negative examples, when the model predicts the negative class but the actual label is positive.

These indices were used to quantitatively evaluate the effectiveness of mangrove extraction, with values closer to 1 indicating better performance.

### 4.2. Comparison of Region Selection and Methods

**Figure 3:**
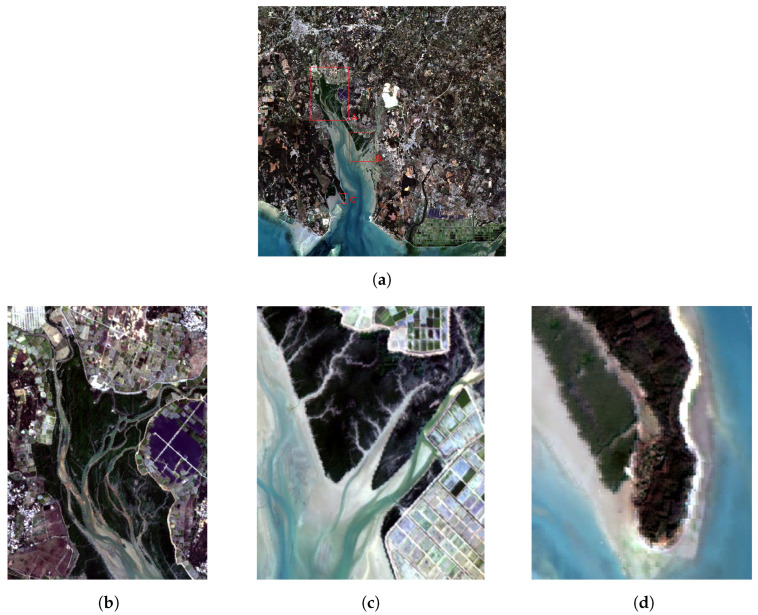
Extraction areas of mangrove samples selected in the Yingluo Port area: (**a**) the locations of the three selected areas in the GF-6 image, indicated by the rectangular boxes labeled A, B, and C; (**b**) area A; (**c**) area B; (**d**) area C.

To ensure the reliability of the experiment, we selected three areas of Yingluo Port for verification and analysis and selected several different extraction algorithms for comparison with the proposed algorithm. In Figure 3, Figure 3a shows the GF-6 image of the Yingluo Port area, with three rectangular boxes labeled A, B, and C representing the areas selected for mangrove target extraction in this study, with enlarged views of these three areas shown in Figure 3b–d. Selecting three areas with differences in both background information and size allows for a better comparative analysis. Among these areas, area A is relatively large and includes more background information such as artificial structures, other vegetation, and aquaculture areas; area B is of medium size and is mainly affected by aquaculture areas and suspended sediments; and area C is relatively small, with a simple background that is mainly influenced by other vegetation.

**Figure 4:**
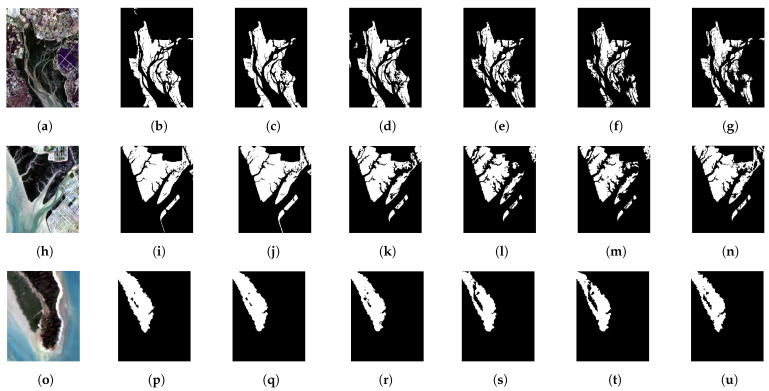
Comparison of extraction results from different algorithms for the three areas: (**a**–**g**) the original image, ground truth map, OMF-WLS extraction map, MF extraction map, CEM extraction map, Adaptive Coherence Estimator (ACE) extraction map, and Maximum Likelihood Estimation (MLE) extraction map for area A; (**h**–**n**) and (**o**–**u**) similarly represent the extraction results of the different algorithms for areas B and C, respectively, in the same order as for area A.

In the comparison results shown in Figure 4, it can be observed that in area A the Constrained Energy Minimization (CEM), Adaptive Coherence Estimator (ACE), and Maximum Likelihood Estimation (MLE) algorithms do not effectively identify the extensive mangrove areas, with significant omissions overall. However, MLE performs better in certain smaller areas. Although MF shows generally better overall identification, there are still misclassifications. The proposed algorithm, while still not effective in some smaller areas, provides better overall extraction results without overly highlighting the gaps between mangroves, and the texture is clear. In area B, due to reduced interference information, none of the algorithms exhibit severe misclassifications. Overall, CEM and ACE still show significant omissions in mangrove detection, especially ACE, which is overly sensitive to spectral information and amplifies differences within the mangrove areas, leading to poor integration. Both MF and MLE demonstrate excellent performance, although some small areas remain unrecognized. The proposed algorithm also performs well at this scale. In area C, with a smaller scale, the overall identification performance of all algorithms improves. However, CEM, ACE, and MLE are excessively sensitive to gaps within the mangroves, resulting in misclassification of sparse mangrove areas as other categories. MF and the proposed algorithm both perform better in this area.

### 4.3. Precision Assessment

The validation data in this paper were processed using ENVI 5.6 and ArcGIS 10.8 software. Through visual interpretation of existing data and high-resolution satellite imagery, mangroves were classified for use as a ground truth map. The data to be validated were processed in MATLAB R2020a software, with various algorithms used to identify and classify the mangroves. The final classification map was then used as the object for accuracy validation.

From Table 2, Table 3 and Table 4, it can be seen that prior to applying the WLS filtering, the performance of the OMF algorithm in area A does not surpass that of the CEM algorithm except for slightly better average accuracy. After applying WLS filtering, however, the edges of the mangroves are enhanced and the internal vegetation density is smoothed. The overall accuracy of the OMF-WLS algorithm improves from 0.95702 to 0.99366 and the Kappa coefficient improves from 0.88436 to 0.98233, with significant improvements in other metrics as well, showing better overall performance.

In area B, while WLS filtering still optimizes the OMF algorithm, the improvement is less pronounced. Although the producer accuracy is lower, the OMF-WLS algorithm still shows superior performance in the other metrics, with all indicators exceeding 96%. Similar to area B, in area C the algorithm performs worse in terms of producer accuracy compared to the CEM and MLE algorithms. In additionally, the average accuracy after WLS filtering decreases by 0.003%. In terms of the other metrics, the proposed algorithm again performs well.

In summary:

- The proposed algorithm shows the best performance in area A and outperforms the other algorithms, with all four metrics exceeding 98%. The CEM algorithm ranks second, with all metrics above 89%.
- In area B, the proposed algorithm in this paper shows the best overall performance, with all metrics above 88%. The ACE algorithm performs best in the PA metric, with the MF algorithm ranking second.
- In area C, the proposed algorithm shows the best overall performance, with all metrics above 91%. The MLE algorithm has the second-best performance.

With regard to the differences in results across different regions, this analysis suggests that the complexity and dynamic changes in spectral features may make it challenging for our OMF-WLS algorithm to accurately separate the spectral signals of mangroves in aquaculture areas where there are numerous artificial structures and reflectors [36]. Additionally, the reflective properties of water bodies may further interfere with the spectral signals of mangroves, increasing the difficulty of extraction. In particular, spectral aliasing becomes more evident in areas with highly reflective artificial structures such as buildings and roads. Shadow effects also lead to variations in local lighting conditions, which in turn affects the extraction of spectral features of mangroves [37]. Of course, tidal changes, seasonal variations, and sediment types are also important environmental variables affecting the spectral characteristics of mangrove ecosystems. These variables cause changes in the spectral signals over different wavelengths by altering the propagation, reflection, and scattering of light in water as well as changes in the physical and chemical properties of plants and sediments. Understanding these impacts is crucial for future use of remote sensing technology to monitor the health status and changing trends of mangrove ecosystems.

Our model demonstrates strong versatility in its ability to extract and recognize spectral features, showcasing robust adaptability across different environments. This adaptability not only validates the model’s robustness under various conditions but also provides technological support for the protection of mangroves worldwide. In the face of different environmental conditions, the proposed model is able to effectively correct for the impact of environmental changes on spectral features through whitening and regularization terms. Specifically, the whitening process eliminates background noise from spectral data, while regularization balances data complexity and model complexity during the training process. Together, these procedures ensure high recognition accuracy across diverse environmental conditions.

In addition to identifying mangroves, our model can also be adapted to play a significant role in environmental monitoring of other ecosystems and for application such as forest health monitoring, wetland ecosystem analysis, and mineral resource exploration. Our model’s generalization capability and high recognition accuracy make it highly promising in these fields.

### 4.4. Discussion

Regarding existing methods, the matched filter performs poorly in environments with a low signal-to-noise ratio (SNR). As the complex background of mangrove environments may result in low SNR, this can affect recognition performance. The CEM algorithm requires solving complex optimization problems, which consumes a large amount of computational resources and may be limited in real-time applications. Additionally, the CEM algorithm is sensitive to noise, which can be complex and diverse in mangrove environments, leading to decreased performance [38]. The ACE algorithm requires extensive experience and precise data for parameter adjustment, and the complexity of the mangrove environment may complicate parameter tuning, impacting recognition effectiveness. The MLE algorithm involves a large amount of statistical computation and optimization, leading to high computational complexity and limited real-time processing capability.

In contrast, the OMF-WLS algorithm presented in this paper not only combines OSP, MF, PCA whitening, and regularization terms, it also integrates the WLS algorithm, which offers significant advantages during the task of mangrove recognition. The WLS algorithm applies a weighted approach to different data points, allowing the model to focus more on reliable signals while reducing the impact of noisy or less significant data. This weighted mechanism is particularly beneficial in complex environments such as mangroves, where data heteroskedasticity and outliers are common. By adaptively adjusting the weights, WLS enhances the accuracy and robustness of signal recognition, making our OMF-WLS algorithm more flexible and stable compared to traditional methods. Furthermore, WLS can effectively handle outliers and noise, ensuring that the final parameter estimation is both precise and reliable. The synergistic combination of OMF and WLS in the proposed algorithm results in superior recognition accuracy, better computational efficiency, and adaptability to complex environments, making it a highly advanced and effective solution for mangrove recognition.

## 5. Conclusions

Although large-scale classification and extraction of remote sensing images have been widely applied in daily life, traditional mangrove identification and extraction methods still have limitations in terms of accuracy and extraction efficiency under complex backgrounds and fail to effectively meet the needs of practical applications. This limitation results in underutilization of time-sensitive remote sensing resources, leading to resource wastage. In this paper, we utilizes target spectral information obtained from remote sensing images and apply the novel OMF-WLS algorithm for mangrove enhancement across backgrounds with different scales. Subsequently, the grayscale images are filtered and Otsu algorithm is used for mangrove extraction.

Compared to traditional methods such as the MF, CEM, and OSP algorithms, the mangrove extraction algorithm proposed in this study performs excellently in a variety of extraction evaluation metrics. The OA value reaches above 0.98 and the Kappa coefficient above 0.94, demonstrating significant advantages. Our algorithm can effectively identify and extract mangroves from various complex backgrounds with high adaptability and precision. Unlike machine learning methods, it only requires traditional mathematical calculation of target spectral information without extensive training, resulting in fast computation and high efficiency. Its rational weighting mechanism is better able to handle complex situations under different backgrounds, reducing misidentification and false positives. The edges of mangroves often have a clear contrast with the surrounding environment, and the WLS algorithm can effectively preserve these edge features while smoothing large areas of mangrove internal information, resulting in clearer and more accurate extraction of mangrove boundaries.

In conclusion, although the algorithm presented in this paper performs better in mangrove identification compared to other methods, it still has limitations in mangrove extraction, including spectral aliasing and band expansion issues. Application of the OMF-WLS algorithm in mangrove conservation can not only provide high-precision capabilities for mangrove identification and monitoring but can also enhance the efficient and scientific protection of mangroves through ecosystem health assessment, biodiversity monitoring, and habitat restoration tracking. This is of great significance for addressing global climate change and protecting marine ecosystems. Future research will focus on technological innovation and method optimization, aiming to provide more powerful technical support for the accurate extraction of mangroves in order to meet the growing needs of ecological monitoring and resource management.

## Figures and Tables

**Table 1:** Feature index after band extension.

Feature Information	Feature Index	Mathematical Expression
Spectral Features	NDVI	$\mathrm{NDVI}=\frac{B4-B3}{B4+B3}$
	EVI	$\mathrm{EVI}=2.5\frac{B4-B3}{B4+6B3-7.5B2+1}$
	NDWI	$\mathrm{NDWI}=\frac{B2-B4}{B2+B4}$
	TSM	$\mathrm{TSM}=0.028B1+0.019B2-5.31\frac{B2}{B1}+0.537$

**Table 2:** Extraction results and metrics from area A in Yingluo Port.

	Area A					
**Metrics**	**OMF-WLS**	**OMF**	**MF**	**CEM**	**ACE**	**MLE**
OA	0.99366	0.95702	0.94834	0.96379	0.90774	0.93233
AA	0.98761	0.95496	0.93621	0.92454	0.81986	0.85944
PA	0.99702	0.87755	0.87412	0.99644	0.93829	0.98994
Kappa	0.98233	0.88436	0.85917	0.89442	0.71469	0.79322

**Table 3:** Extraction results and metrics from area B in Yingluo Port.

	Area B					
**Metrics**	**OMF-WLS**	**OMF**	**MF**	**CEM**	**ACE**	**MLE**
OA	0.98256	0.97988	0.95222	0.90389	0.90925	0.94142
AA	0.98161	0.97975	0.93275	0.85014	0.85781	0.90857
PA	0.96678	0.95847	0.96839	0.99611	0.99997	0.99838
Kappa	0.96000	0.95397	0.88727	0.75997	0.77409	0.85839

**Table 4:** Extraction results and metrics from area C in Yingluo Port.

	Area C					
**Metrics**	**OMF-WLS**	**OMF**	**MF**	**CEM**	**ACE**	**MLE**
OA	0.98682	0.98644	0.94060	0.96824	0.92683	0.97953
AA	0.96329	0.96332	0.94219	0.89635	0.85074	0.93159
PA	0.98095	0.97773	0.73395	0.99179	0.76160	0.99959
Kappa	0.94696	0.94552	0.79087	0.86376	0.70891	0.91461

## Data Availability

Data are contained within the article.
